# Automatic Hip Detection in Anteroposterior Pelvic Radiographs—A Labelless Practical Framework

**DOI:** 10.3390/jpm11060522

**Published:** 2021-06-07

**Authors:** Feng-Yu Liu, Chih-Chi Chen, Chi-Tung Cheng, Cheng-Ta Wu, Chih-Po Hsu, Chih-Yuan Fu, Shann-Ching Chen, Chien-Hung Liao, Mel S. Lee

**Affiliations:** 1 Compal Electronics, Smart Device Business Group, Taipei 114, Taiwan; ryk_liu@compal.com; 2 Department of Physical Medicine and Rehabilitation, Chang Gung Memorial Hospital, Chang Gung University, Linkou, Taoyuan 333, Taiwan; claudia5477@gmail.com; 3 Department of Trauma and Emergency Surgery, Chang Gung Memorial Hospital, Chang Gung University, Linkou, Taoyuan 333, Taiwan; atong89130@gmail.com (C.-T.C.); m7831@cgmh.org.tw (C.-P.H.); drfu5564@yahoo.com.tw (C.-Y.F.); 4 Center for Artificial Intelligence in Medicine, Chang Gung Memorial Hospital, Linkou, Taoyuan 333, Taiwan; 5 Department of Orthopedic Surgery, Kaohsiung Chang Gung Memorial Hospital, Kaohsiung 833, Taiwan; oliverwu429@cgmh.org.tw (C.-T.W.); mellee@cgmh.org.tw (M.S.L.)

**Keywords:** deep learning, hip detection, deep convolutional neural network, radiography

## Abstract

Automated detection of the region of interest (ROI) is a critical step in the two-step classification system in several medical image applications. However, key information such as model parameter selection, image annotation rules, and ROI confidence score are essential but usually not reported. In this study, we proposed a practical framework of ROI detection by analyzing hip joints seen on 7399 anteroposterior pelvic radiographs (PXR) from three diverse sources. We presented a deep learning-based ROI detection framework utilizing a single-shot multi-box detector with a customized head structure based on the characteristics of the obtained datasets. Our method achieved average intersection over union (IoU) = 0.8115, average confidence = 0.9812, and average precision with threshold IoU = 0.5 (AP50) = 0.9901 in the independent testing set, suggesting that the detected hip regions appropriately covered the main features of the hip joints. The proposed approach featured flexible loose-fitting labeling, customized model design, and heterogeneous data testing. We demonstrated the feasibility of training a robust hip region detector for PXRs. This practical framework has a promising potential for a wide range of medical image applications.

## 1. Introduction

The deep convolutional neural network (DCNN) has shown a significant breakthrough in many aspects of commercial image differentiation and identification. In recent years, DCNNs have also played important roles in medical image analysis [1,2]. For example, the ChestX-ray8 [3] and MURA [4] are two representative studies utilizing the state-of-the-art DCNN classification and visualization models to detect and locate disease patterns in the chest and musculoskeletal radiographs.

Some studies employ a more delicate “two-step” classification strategy, which first detects specific ROIs [5,6,7,8,9], followed by conventional classification methods [10,11,12]. A seminal work is the automatic knee osteoarthritis diagnosis in lateral knee radiographs, where knee regions are first identified [13], followed by classification and heatmap visualization [14]. The advantage of this “two-step” approach is the capability to identify subtle localized abnormalities and has gradually become the mainstream technology, especially for the analysis of PXRs, including fracture subclass identification [15], hip osteoarthritis grading [16], and avascular necrosis detection [17]. Nonetheless, the above studies barely mentioned the model parameter settings and selection criteria, and none of them reported the confidence score for the detected ROIs, which the confidence score is a crucial metric indicating the likelihood that the predicated ROI contains the correct object.

A critical component for a successful “two-step” classification system is accurate ROI detection, which falls into computer vision object detection tasks [18], usually tackled by different strategies [19]. Among these methods, the bounding-box-based methodology is advantageous for its lower annotation workload and simple implementation, which is proven to be effective in popular computer vision applications in other sectors. In order to identify multiple objects across different scales in one image, one must generate anchor boxes of varied sizes and aspect ratios for hyper-parameter optimization. However, there is usually a small number of non-overlapping objects in medical images. It is not optimal to apply the same object detection parameters on different underlying applications.

In this work, we propose a labor-less practical framework of ROI detection and parameter selection in medical images. To the best of our knowledge, this is the first work that provides a systematic guideline for parameter selection based on the obtained datasets and has a promising potential for a wide range of medical image applications for further personalized medicine.

## 2. Materials and Methods

### 2.1. Dataset Acquisition

This retrospective study analyzed hip joints seen on 7399 PXRs from three diverse sources, including the Chang Gung Memorial Hospital Osteoarthritis (CGOA) dataset containing 4290 high-resolution radiographs, the second Osteoarthritis Initiative Hip (OAIH, pelvic radiograph dataset extracted from a subset of data from the OAI [20]) dataset containing 3008 radiographs with relatively lower resolutions, and the third Google Image Search (GIS) dataset containing 101 heterogeneous radiographs. Table 1 lists the summary statistics of these datasets. This experimental design, which utilizes radiographs generated from diverse sources of different imaging protocols, resolutions, and ethnicities, ensures that model generalization can be achieved. Details of these three datasets can be found in Table 1.

### 2.2. Data Annotation

**Figure 1:**
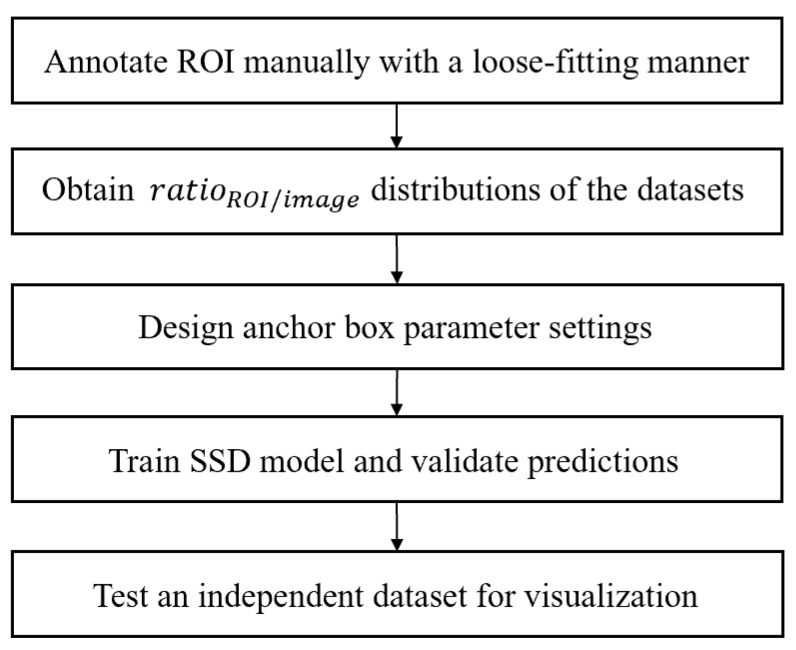
Overview of the proposed framework for hip ROI detection.

Figure 1 shows the overview of the proposed framework.

Clinical readings on etiology and grading of all CGOA images were performed by one physician with 15 years of clinical experience. To annotate hip regions of interest, we employed three annotators trained to place square bounding boxes approximately centered at the femoral head or the artificial hip joint with customized GUI software. It is noted that identifying a complete round femoral head in healthy hips is relatively straightforward; however, for cases with disrupted hip conditions with collapsed femoral heads, we employed a loose-fitting manner to make sure every hip joint lay appropriately in the bounding box. All the labeled ROIs in the CGOA dataset were visually reviewed by physicians, and the ROI annotators used the same rules to annotate the remaining OAIH and GIS datasets.

### 2.3. Proposed SSD Model Architecture for ROI Detection in Hip Radiographs

**Figure 2:**
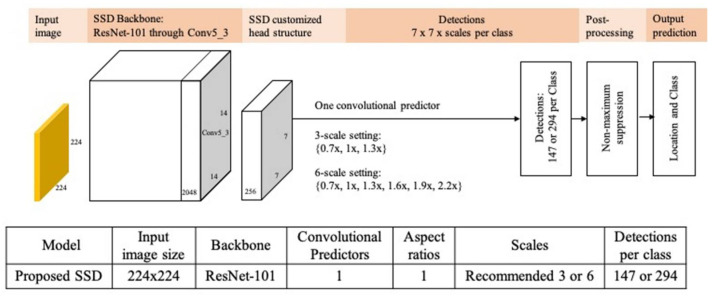
Comparison of SSD model architectures. Proposed architecture with ResNet-101 backbone and other customized settings.

The proposed hip region detection architecture simplifies existing SSD model architecture (as Figure 2) [9], which was originally developed for detecting multiple objects with different sizes and aspect ratios in applications. 

For ROI detection in medical images, we replaced the SSD VGG-16 backbone by ResNet-101 [11] backbone, which was pre-trained on ImageNet [21]. All these modifications could reduce ROI detections from several thousands to a few hundreds, decreasing training time and complexity as well as increasing detection accuracy and confidence.

**Figure 3:**
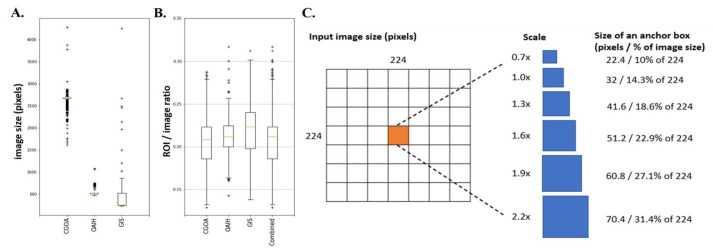
Comparison of three radiographic dataset distributions and generation of anchor boxes. (**A**) The image size distributions of the three datasets. (**B**) The distributions of three datasets. (**C**) Generation of anchor boxes for the one feature layer of the customized SSD head structure. With an input square image with 224 × 224 pixels, there are 7 × 7 grid cells with 32 × 32 pixels with scale = 1, and each grid cell can use different scale parameters to generate various sizes of anchor boxes covering 10% to 31.4% of the input image size, depending on the training image size distributions.

To best determine the anchor box parameter settings, we first defined the size of the square ROI divided by the length of the long side of the input image (zero padding to a square if needed). This ratio is designed as a normalizer, making the anchor boxes and ROI instances compatible across different datasets. Next, we analyzed image size distributions (Figure 3A) and distributions (Figure 3B) of the three available heterogeneous datasets, where the ratios lie mostly between 10% to 30%.

We specified the input image size of 224 × 224 pixels split by 7 × 7 grid cells, where each grid cell is of size 32 × 32 pixels. We set 6 equally spaced scales parameters {0.7, 1.0, 1.3, 1.6, 1.9, 2.2} (Figure 3C) so that the smallest and largest anchor boxes could cover 10% and 31.4% of the images, respectively. This design ensures that the designed anchor boxes can identify appropriate hip ROIs in the datasets.

### 2.4. Data Preprocessing, Training, and Evaluation

For data preprocessing, each radiograph was zero padding to a square image and resized to 224 × 224 pixels with 8-bit grayscale before feeding into the model. The model was implemented by fastai v0.7 library [22] with Python 3.6.4, and we randomly split the combined CGOA and OAIH dataset into 90% for training and 10% for validation once, and used all 101 GIS radiographs as the independent test dataset. We fixed the same training and validation images in either the combined dataset or each individual dataset in all experiments for fair comparison. For evaluation, we used the standard IoU metric for comparing the predicted bounding box *B_pred_* and ground truth bounding box *B_gt_*:

$$\mathrm{IoU}=\frac{B_{pred}\cap B_{gt}}{B_{pred}\cup B_{gt}}$$

where ∩ and ∪ denote intersection and union, respectively. We reported the associated confidence, which denotes the likelihood that the anchor box contains an object, for each predicted bounding box, average IoU, average confidence, minimal confidence, and AP50, as the 0.5 cutoff indicates poor ROI detection, which may cause issues for downstream analysis.

## 3. Results

### 3.1. Demographics of the Study Population

The original CGOA cohort contained 4643 high resolution radiographs, including 3013 patients who underwent hip surgery with an average age of 63.06 ± 15.72 years and 40.8% being male, and 1630 control cases from emergency room without undergoing hip surgery with an average age of 44.88 ± 20.46 years and 68.2% being male. Among the 3013 surgical patients, 353 cases with severe fractures were excluded due to completely different morphology and treatment options. The remaining 2660 trauma patients including hundreds of occult fracture cases and 1630 control cases constructed the COGA dataset. The second OAIH dataset was a consolidated pelvic radiograph dataset extracted from subset of data from the OAI project, which recruited 4796 participants from February 2004 to May 2006 to form a baseline cohort (58% female and ranged in age from 45 to 79 years at time of recruitment). The third GIS dataset was acquired through Google image search engine, and the demographics are not available.

### 3.2. Model Performance and Visualization

In Table 2, we take a closer look at the best performance results and carefully examine those cases where hip ROIs had IoU < 0.5. As AP50 metrics were 1 in both training and validation set and 0.9901 in the independent GIS test set, we only identified two cases below IoU 0.5 cutoff, which may indicate poor ROI detection and cause issues for downstream analysis. 

**Figure 4:**
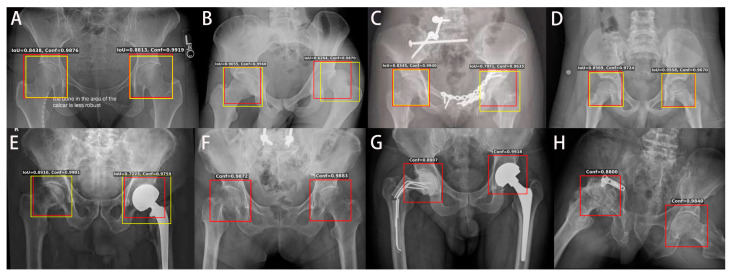
Visualization of hip ROI detection results on the testing dataset. Yellow boxes indicate manual labels, and red boxes indicate detected hip ROIs. In all scenarios, the ROI could be detected well in both hips. (**A**) A radiograph with some text outside the key hip area. (**B**) A radiograph with left hip dislocation. (**C**) A radiograph with plates on the left pubic ramus and acetabulum. The hips were detected correctly. (**D**) A radiograph of pediatric patients. (**E**) A radiograph showing left hip replacement and deformity of the right hip. (**F**) Right proximal femoral fracture. (**G**) The right hip showing a fracture of a temporal cemented prosthesis and left total hip replacement. (**H**) Right acetabular fixation with plate with destructed femoral head.

We further examined other radiographs in the heterogeneous test set, and the hip ROI detection showed several representative results, as Figure 4 presents. Figure 4A shows a radiograph with some text outside the key hip area. Figure 4B shows the dislocation on the left hip, but the detected hip ROI covers most key features of the left hip. Figure 4C shows a radiograph with plates on the left pubic ramus and acetabulum, and ROI can detect the hips correctly. Figure 4D shows a radiograph with pediatric patients. Figure 4E shows left hip artificial can be detected correctly. Figure 4F hip ROI indicated right proximal femoral fracture. Figure 4G shows right temporal cemented prosthesis fracture and left total hip replacement, and the hip ROI can be detected. Finally, as shown in Figure 4H, the hip ROI was able to detect right acetabular fracture with plate fixation and destructed femoral head. These results suggest that our model with specially designed anchors and trained by diverse datasets is a general and robust hip region detector that can be applicable for a wide range of heterogeneous datasets with different qualities and resolutions and can be potentially useful for automated assessment of many hip bone conditions.

## 4. Discussion

In this work, we have demonstrated a practical framework for detecting regions of interest in medical images. With the case study for hip detection in PXRs, we achieved average IoU over 80% and average confidence higher than 95%. These independent test set showed promising ROI detection results on GIS with heterogeneous resolutions and appearance. The proposed hip region detection architecture simplified existing SSD model architecture, which was originally developed for detecting multiple objects with different sizes and aspect ratios in applications. For ROI detection in medical images, there are usually one or two important organs in one radiograph. It is feasible to have a simplified SSD architecture with only one feature layer as the only convolutional predictor, with an appropriate receptive field size, one aspect ratio (1:1 in for hip ROI), and a small set of scales.

Compared to traditional object detection tasks, which need to recognize multiple objects with different sizes and aspect ratios in images and videos, the proposed SSD architecture has the advantages of simpler structure, higher IoU accuracy, and reliable confidence. The challenge of determining those empirical parameter settings now relies on the basic statistics on the available datasets to generate enough anchor boxes. Our results suggest that more anchors do not necessarily encourage higher IoU but may decrease the prediction performance. The proposed method provides a more effective approach for anchor design and parameter optimization.

Annotation by doctors is time-consuming and is usually the bottleneck for medical image analysis. The approximate identification of hip regions by automated and accurate ROI detection is critical for automated computer-assisted analysis for screening and diagnostics. The proposed framework provides a guideline for parameter settings in anchor-based object detection algorithms, and it is especially useful for applications such as joint identification in medical image problems. Several studies have reported good results [14,15,16,17]. However, heavy labeling workload and cost of physicians’ label are another consideration that has limited this method from going global. Our study provided a method of manual annotation with approximation identification of hip regions that can be performed effectively and inexpensively.

Medical artificial intelligence is progressive in order to change the healthcare system, and various DCNNs have showed that it is feasible to detect lesions from pathologic images [23] and radiography [24]. These algorithms presented outstanding achievement in disease detection or prediction of whose performance is not inferior to that of the physicians [23,24,25]. These results inspire us in that DCNN might help individuals in the healthcare sector in different ways. However, the development of medical AI is not accessible due to some limitations. The data clearance and accurate label were considered fundamental for deep learning because of the limited size and data quality of medical images [19] and the high cost of a medical expert to perform labeling [26]. Moreover, the hip ROI detection system can help the physician to label the lesion in a weak supervision way, wherein we can pick out the hip regions and save time for the physician to crop and copy the images. The reduction of the barrier between an outliner and the way in which to attract more physicians and scientists to join a new rising technologic field are other issues to be considered in the real world. In this study, we developed the diagnostic assistance system and created a useful tool for reducing the workload during data collection and tuning. With our tool, we can simply label workload, minimize the calculation requirement, and eventually make the physician use it in the way they need. There are numerous existing programs [27,28] that can help orthopedics to plan the surgical strategy. Our algorithm might accelerate the speed of these programs by reducing calculation requirements in the future. The utility of such ROI detection approaches highly depends on the downstream applications. With input of clinical physicians’ expertise, this automated hip ROI detection enables applications such as fracture identification, osteoarthritis assessment, osteoporosis, and even surgical prediction in the future. The evaluations of such applications and integrated systems remain to be investigated in future works and remain to be open research topics.

## 5. Limitation

Our study provided a feasible framework of automated ROI labeling. However, there are still some limitations in the existing method. First, the manual hip annotation with loose-fitting criteria is not unique and can be varied from person to person, especially for those cases with destructed hips. In these situations, a closer visual examination is needed. Because of the data distribution, we excluded most images from patients with endomedullary prostheses to make the training data solid. Therefore, we did not have these kinds of images for further validation, which might impact the usability of this algorithm. Lastly, limited medical image data might influence the performance of this algorithm. Increasing data from other sources might increase the performance and prevent the possibility of overfitting.

## 6. Conclusions

In conclusion, with the proposed DCNN framework, we can identify the hip joint with high accuracy, reliability, and reproducibility. It has a clear approach for ROI detection in plain X-ray and has practical usefulness for future applications in medical imaging. Increasing data and destructed hip analysis might improve the performance of this algorithm. However, the downstream application of hip ROI detection is a further research direction, and with our tool, we can simply label workload and eventually adjust the algorithm to fulfil the physicians’ need to achieve the aim of personalized healthcare.

## Figures and Tables

**Table 1:** Summary statistics of the three datasets used in this study.

Datasets	Number of Images	Max (Pixels)	Min (Pixels)	Median (Pixels)	Mean (Pixels)	Standard Deviation	Recruit Year
CGOA	4290	4280	1616	2688	2635.8	201.1	2008–2017
OAIH	3008	1080	466	535	571.3	97.0	2004–2014
GIS	101	4256	225	258	515.3	626.6	N/A

**Table 2:** Detailed performance metrics with the optimal parameters using the proposed hip region detection architecture.

Datasets	Number of Images	Number of Hip ROIs	Avg IoU	Avg Confidence	Minimal IoU	Number of Hip ROIs with IoU < 0.5	AP50
All: CGOA & OAIH & GIS	7399	14,798	0.9176	0.9688	0.3861	2	0.9999
Train: 90% CGOA & OAIH	6568	13,136	0.9260	0.9698	0.5955	0	1
Valid: 10% CGOA & OAIH	730	1460	0.8571	0.9582	0.5907	0	1
Test: GIS	101	202	0.8115	0.9812	0.3861	2	0.9901

## Data Availability

The data are partially available under the request of the audience.

## References

[1] Cheng C.-T., Ho T.-Y., Lee T.-Y., Chang C.-C., Chou C.-C., Chen C.-C., Chung I.-F., Liao C.-H. (2019). Application of a deep learning algorithm for detection and visualization of hip fractures on plain pelvic radiographs. Eur. Radiol..

[2] Xue Y., Zhang R., Deng Y., Chen K., Jiang T. (2017). A preliminary examination of the diagnostic value of deep learning in hip osteoarthritis. PLoS ONE.

[3] Wang X., Peng Y., Lu L., Lu Z., Bagheri M., Summers R.M. Chest X-ray 8: Hospital-scale chest X-ray database and benchmarks on weakly-supervised classification and localization of common thorax diseases. Proceedings of the IEEE Conference on Computer Vision and Pattern Recognition.

[4] Rajpurkar P., Irvin J., Bagul A., Ding D., Duan T., Mehta H., Yang B., Zhu K., Laird D., Ball R.L. (2017). MURA: Large dataset for abnormality detection in musculoskeletal radiographs. arXiv.

[5] Redmon J., Farhadi A. (2018). YOLOv3: An incremental improvement. arXiv.

[6] Redmon J., Divvala S., Girshick R., Farhadi A. You only look once: Unified, real-time object detection. Proceedings of the IEEE Conference on Computer Vision and Pattern Recognition.

[7] Redmon J., Farhadi A. (2016). YOLO9000: Better, Faster, Stronger. arXiv.

[8] Bochkovskiy A., Wang C.-Y., Liao H.-Y.M. (2020). YOLOv4: Optimal speed and accuracy of object detection. arXiv.

[9] Liu W., Anguelov D., Erhan D., Szegedy C., Reed S., Fu C.-Y., Berg A.C. (2016). SSD: Single shot multibox detector. Proceedings of the Computer Vision—ECCV 2016.

[10] Simonyan K., Zisserman A. (2014). Very deep convolutional networks for large-scale image recognition. arXiv.

[11] He K., Zhang X., Ren S., Sun J. Deep residual learning for image recognition. Proceedings of the IEEE Conference on Computer Vision and Pattern Recognition.

[12] Xie S., Girshick R., Dollár P., Tu Z., He K. Aggregated residual transformations for deep neural networks. Proceedings of the IEEE Conference on Computer Vision and Pattern Recognition.

[13] Tiulpin A., Thevenot J., Rahtu E., Saarakkala S. (2017). A novel method for automatic localization of joint area on knee plain radiographs. Proceedings of the Image Analysis.

[14] Tiulpin A., Thevenot J., Rahtu E., Lehenkari P., Saarakkala S. (2018). Automatic knee osteoarthritis diagnosis from plain radiographs: A deep learning-based approach. Sci. Rep..

[15] Krogue J.D., Cheng K.V., Hwang K.M., Toogood P., Meinberg E.G., Geiger E.J., Zaid M., McGill K.C., Patel R., Sohn J.H. (2020). Automatic hip fracture identification and functional subclassification with deep learning. Radiol. Artif. Intell..

[16] von Schacky C.E., Sohn J.H., Liu F., Ozhinsky E., Jungmann P.M., Nardo L., Posadzy M., Foreman S.C., Nevitt M.C., Link T.M. (2020). Development and validation of a multitask deep learning model for severity grading of hip osteoarthritis features on radiographs. Radiology.

[17] Li Y., Li Y., Tian H. (2020). Deep learning-based end-to-end diagnosis system for avascular necrosis of femoral head. IEEE J. Biomed. Health Inform..

[18] Zhao Z.-Q., Zheng P., Xu S.-T., Wu X. (2019). Object detection with deep learning: A review. IEEE Trans. Neural Netw. Learn. Syst..

[19] Esteva A., Chou K., Yeung S., Naik N., Madani A., Mottaghi A., Liu Y., Topol E., Dean J., Socher R. (2021). Deep learning-enabled medical computer vision. NPJ Digit. Med..

[20] Joseph G.B., Hilton J.F., Jungmann P.M., Lynch J.A., Lane N.E., Liu F., McCulloch C.E., Tolstykh I., Link T.M., Nevitt M.C. (2016). Do persons with asymmetric hip pain or radiographic hip OA have worse pain and structure outcomes in the knee opposite the more affected hip? Data from the Osteoarthritis Initiative. Osteoarthr. Cartil..

[21] Russakovsky O., Deng J., Su H., Krause J., Satheesh S., Ma S., Huang Z., Karpathy A., Khosla A., Bernstein M. (2015). ImageNet large scale visual recognition challenge. Int. J. Comput. Vis..

[22] Howard J., Gugger S. (2020). Fastai: A layered API for deep learning. Information.

[23] Esteva A., Kuprel B., Novoa R.A., Ko J., Swetter S.M., Blau H.M., Thrun S. (2017). Dermatologist-level classification of skin cancer with deep neural networks. Nature.

[24] Cheng C.-T., Wang Y., Chen H.-W., Hsiao P.-M., Yeh C.-N., Hsieh C.-H., Miao S., Xiao J., Liao C.-H., Lu L. (2021). A scalable physician-level deep learning algorithm detects universal trauma on pelvic radiographs. Nat. Commun..

[25] Gulshan V., Peng L., Coram M., Stumpe M.C., Wu D., Narayanaswamy A., Venugopalan S., Widner K., Madams T., Cuadros J. (2016). Development and validation of a deep learning algorithm for detection of diabetic retinopathy in retinal fundus photographs. JAMA.

[26] Tobore I., Li J., Yuhang L., Al-Handarish Y., Kandwal A., Nie Z., Wang L. (2019). Deep learning intervention for health care challenges: Some biomedical domain considerations. JMIR Mhealth Uhealth.

[27] Meermans G., Malik A., Witt J., Haddad F. (2011). Preoperative radiographic assessment of limb-length discrepancy in total hip arthroplasty. Clin. Orthop. Relat. Res..

[28] Schröter S., Ihle C., Mueller J., Lobenhoffer P., Stöckle U., van Heerwaarden R. (2013). Digital planning of high tibial osteotomy. Interrater reliability by using two different software. Knee Surgery Sports Traumatol. Arthrosc..

